# Colonization of a Central Venous Catheter by the Hyaline Fungus *Fusarium solani* Species Complex: A Case Report and SEM Imaging

**DOI:** 10.1155/2013/618358

**Published:** 2013-07-02

**Authors:** Alberto Colombo, Giuseppe Maccari, Terenzio Congiu, Petra Basso, Andreina Baj, Antonio Toniolo

**Affiliations:** ^1^Laboratory of Medical Microbiology, Department of Clinical and Experimental Medicine, University of Insubria and Ospedale di Circolo e Fondazione Macchi, 21100 Varese, Italy; ^2^Laboratory of Human Morphology “L. Cattaneo”, Department of Morphology and Surgery, University of Insubria, 21100 Varese, Italy; ^3^Forensic Institute, Department of Life Sciences, University of Insubria, 21100 Varese, Italy

## Abstract

The incidence of opportunistic infections by filamentous fungi is increasing partly due to the widespread use of central venous catheters (CVC), indwelling medical devices, and antineoplastic/immunosuppressive drugs. The case of a 13-year-old boy under treatment for acute lymphoblastic leukemia is presented. The boy was readmitted to the Pediatric Ward for intermittent fever of unknown origin. Results of blood cultures drawn from peripheral venous sites or through the CVC were compared. CVC-derived bottles (but not those from peripheral veins) yielded hyaline fungi that, based on morphology, were identified as belonging to the *Fusarium solani* species complex. Gene amplification and direct sequencing of the fungal ITS1 rRNA region and the EF-1alpha gene confirmed the isolate as belonging to the *Fusarium solani* species complex. Portions of the CVC were analyzed by scanning electron microscopy. Fungi mycelia with long protruding hyphae were seen into the lumen. The firm adhesion of the fungal formation to the inner surface of the catheter was evident. In the absence of systemic infection, catheter removal and prophylactic voriconazole therapy were followed by disappearance of febrile events and recovery. Thus, indwelling catheters are prone to contamination by environmental fungi.

## 1. Introduction

Opportunistic fungal pathogens can enter the body via airways, skin at the site of tissue breakdown, and mucosal membranes. In compromised patients, the incidence of infections by filamentous fungi is increasing due to the mounting use of central venous catheters (CVC), other indwelling medical devices, and treatments with antineoplastic and immunosuppressive drugs that, possibly, damage mucosae and reduce immune defenses. Broad-spectrum antibacterial treatment may also represent a risk factor for opportunistic fungal infections [1]. In particular, disseminated infections by *Fusarium* spp.—a fast-growing, angioinvasive, immunosuppressive, and adhesive opportunistic fungal pathogen—have been reported in patients with hematologic malignancies and are associated with poor outcome [2]. Treatment with glucocorticoids predisposes patients to fusariosis mainly via impairment of the anticonidial macrophage function [3]. In these cases, prompt detection of fungal colonization and identification of the responsible organism are critical for initiating appropriate treatment. In fact, inappropriate therapy is associated with bloodstream dissemination and systemic disease [4].

## 2. Methods

### 2.1. Patient

The case of a 13-year-old boy diagnosed with acute lymphoblastic leukemia at the Pediatric Unit of the Ospedale di Circolo in Varese (Italy) is presented.

### 2.2. Clinical Microbiology Methods

Blood cultures (BACTEC Plus Aerobic/F* and Plus Anaerobic/F* bottles; BD, Buccinasco, Italy) have been performed, according to the manufacturer's indications, using a 7-day incubation protocol plus an additional 7-day incubation period at 35°C. Sabouraud dextrose agar plates (Oxoid, Rodano, Italy) were incubated in aerobic atmosphere at 35°C for 7 days.

### 2.3. Molecular Methods

**Figure 1:**
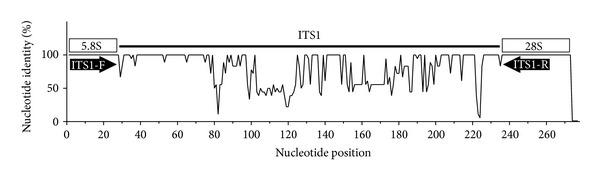
Percent nucleotide identity in the ITS1 rRNA region of isolates* of Fusarium *species based on sequences deposited in the NCBI database. Novel oligonucleotide primers have been designed on the basis of conserved parts of the 5.8 S rRNA region (primer ITS1-F: CGAATCTTTGAACGCACAT) and of the 28S rRNA region (primer ITS1-R: TAAGTTCAGCGGGTATTCCTAC). Sequences of the ITS1 region are variable among different isolates.

For obtaining genomic DNA from fungal isolates, portions of three different colonies grown in Sabouraud dextrose agar plates were suspended in 250 *μ*L of chitinase buffer (0.50 mM phosphate buffer, pH 6.1 plus 1.6 U/mL bacterial chitinase; Sigma, St. Louis, MO, USA) and incubated at 55°C for 1 hr followed by 1 hr incubation at 56°C with 180 *μ*L of lysis buffer plus 20 *μ*L of proteinase K (Qiagen, Milan, Italy). DNA was purified using QIAmp spin columns (Qiagen). Molecular tests were performed in triplicate. Custom oligonucleotide primers were synthesized by Sigma-Aldrich (London, UK). A generic primer pair for amplification of the fungal 18S rRNA gene was used for preliminary identification of the isolate [5]. A novel primer pair specific for the ITS1 rRNA region was designed based on multiple alignments of the rRNA genomic fragments of pathogenic and environmental* Fusarium *spp. using different bioinformatic tools (*COMPASSS* [6]; *CLC bio*, Aarhus, Denmark) (Figure 1). Amplicons were also obtained for the elongation factor-1 alpha (EF-1 alpha) gene using the primers and conditions reported by Wang et al. [7]. Templates were amplified for 32 cycles (30 sec annealing and extension times) using AmpliTaq Gold DNA polymerase with buffer II (Life Technologies, Monza, Italy). PCR products were visualized by agarose gel electrophoresis using the Lonza FlashGel Fast 1.2% Agarose Gel System (EuroClone, Pero, Italy). Two ATCC strains (MYA-3636 and ATCC-90862) were used as references for the identification of *F. solani* species complex. Amplicons were sequenced directly using BigDye Terminator V1.1 reagents (Life Technologies) and an ABI automated sequencer. The BLAST program was used for identifying fungal sequences in databases of the National Center for Biotechnology Information (NCBI, Bethesda, MD, USA) and of *Fusarium* MLST (http://www.cbs.knaw.nl/fusarium/).

### 2.4. Scanning Electron Microscopy (SEM)

The CVC removed from the patient was washed once in phosphate buffered saline ((PBS) pH 7.2), then fixed in 4% paraformaldehyde for 1 hr. In order to expose the lumen, the catheter was split longitudinally with a razor blade. Specimens were postfixed in a solution of 1% osmium tetroxide and 1.25% potassium ferrocyanide for 2 hr. Specimens were washed in PBS, dehydrated in ascending grades of ethanol, subjected to critical point drying in CO_2_, and coated with 10 nm of pure gold in a vacuum sputter coater (Emitech K550, Ashford, UK). Specimens were observed in direct mode (SE) and back scattered electron (BSE) mode using a Philips XL 30 (Philips/FEI, Eindhoven, Holland) scanning electron microscope field emission gun.

## 3. Results

A 13-year-old boy diagnosed with acute lymphoblastic leukemia was immediately started with treatment (prednisone, dexamethasone, vincristine, asparaginase, and daunorubicin) via a central vascular catheter ((CVC), Broviac). One month later, the patient was re-admitted to the Pediatric Unit for intermittent fever of unknown origin. Blood cultures were performed once a day for 6 days by drawing blood directly through the CVC and from peripheral venous sites. After 2-3 days of incubation, direct microscopy of blood culture medium revealed filamentous fungi in bottles seeded with blood taken from CVC, but not in bottles seeded with blood taken from venous sites. No bottles from peripheral veins were positive after 14-day incubation. After 5-6 days of incubation, Sabouraud dextrose agar medium seeded with CVC-derived blood cultures yielded colonies of hyaline fungi. Cottony colonies with white aerial mycelium, and a creamy reverse were seen. Microscopic examination showed hyaline and septate hyphae, simple conidiophores, and branched monophialides. Macroconidia were moderately curved, stout, thick-walled 3–5 septate, 4–6 *μ*m in diameter and up to 65 *μ*m in length. Microconidia elongated from long monophialides and were one celled. No Chlamydoconidia were present. Based on morphologic properties, the fungus was identified as *F. solani *species complex.

Identification was confirmed by molecular tests. Amplicons produced using generic 18S rRNA primers provided information on the fungal genus, whereas amplicons obtained with primers specific for the ITS1 rRNA region [8] as well as the EF-1 alpha gene [7] confirmed the isolate as belonging to the *F. solani* species complex. Six days after performing blood cultures, detection of a fungal pathogen (preliminarily identified as *F. solani*) in blood samples taken through the CVC (but not in those taken from peripheral veins) prompted CVC removal. Oral prophylactic treatment with voriconazole was started (200 mg q 12 hr for 5 days, followed by 100 mg q 12 hr for 12 days).

**Figure 2:**
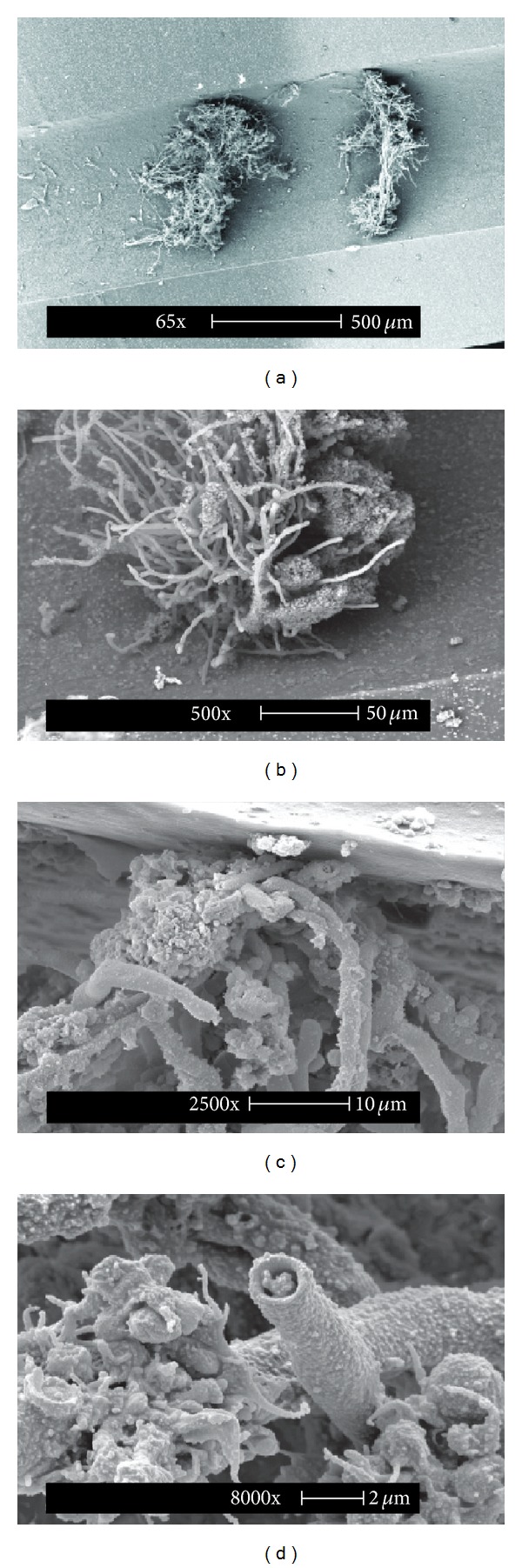
Scanning electron microscopy images of the tip and cuff of the central venous catheter removed from a child with intermittent fever of unknown origin. (a) Mycelial formation in the catheter lumen. Of note is the long fungal protrusion into the lumen. ((b) and (c)) Magnifications show adhesion of the fungal formation to the inner surface of catheter. (d) Detail of the aerial mycelium.

Tip and cuff of the removed CVC were cultured separately in Sabouraud dextrose agar plates. After 6-day incubation, fungal colonies developed. Again, morphological and molecular analysis identified the isolate as *F. solani *species complex. To investigate catheter colonization, CVC portions were prepared for scanning electron microscope (SEM) according to published methods [9]. SEM documented the adhesion of *F. solani* mycelia to the inner CVC wall (Figure 2). In the absence of positive blood cultures from peripheral venous sites, catheter removal and prophylactic antifungal therapy resulted in the disappearance of febrile events. The patient was discharged 30 days later and successfully completed chemotherapy at home.

## 4. Discussion

*Fusarium* species are cosmopolitan soil saprophytes and facultative plant pathogens that spread mainly through air and may cause infection or toxicosis in humans and animals [9]. The *F. solani *species complex comprises at least five members that occasionally are responsible of a wide range of human infections, especially in compromised hosts [10, 11].

This clinical report shows that classical microbiology, together with molecular methods, may allow prompt identification of fungal pathogens in indwelling catheters of compromised patients. In addition, even if not applicable to routine diagnosis, SEM analysis may provide relevant information on fungal adhesion, growth, and colonization. In the reported case, SEM revealed proliferative mycelia formations firmly adhering to the inner wall of the catheter. The study illustrates the possibility of prolonged colonization of a CVC by hyaline fungi.

CVC-related complications may occur in up to 30% of children with hematologic diseases [12]. In the reported case, removal of the colonized CVC and antifungal therapy were followed by prompt remission. In other cases, systemic infections occurred even after removal of the colonized CVC [13]. The need for meticulous care of long-term catheters is emphasized by this case. The report also points to the necessity of developing catheter materials that may resist fungal adhesion and colonization.

In conclusion, innovative ways for detecting and identifying fungi in clinical samples are starting to complement classical microbiology and may improve prognosis in compromised patients [14].
